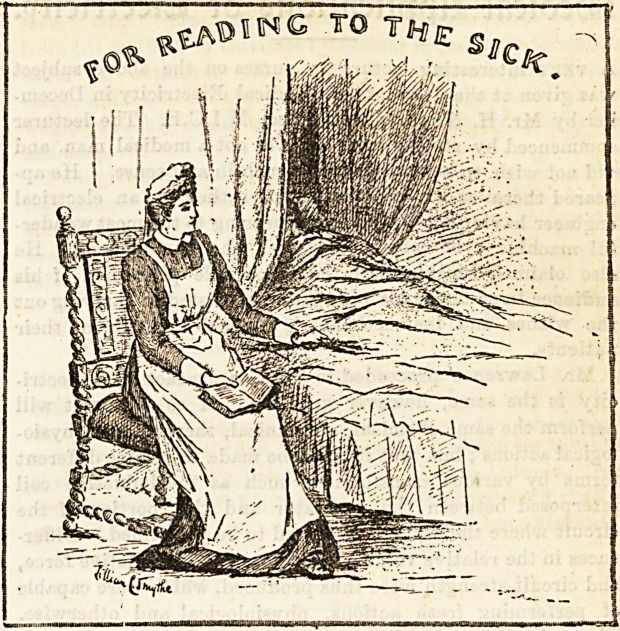# Extra Supplement—The Nursing Mirror

**Published:** 1891-01-24

**Authors:** 


Being the Extra Nursing Supplement of "The Hospital" Newspaper.

Contributions for this Supplement should be addressed to the Editor, The Hospital, 140, Strand, London, W.C., and should have the word "Nursing" plainly written in left-hand top corner of the envelope.

### En Passant.

MURRAY ROYAL ASYLUM.—A fancy dress ball, at which some 130 were present, was held at James Murray's Royal Asylum, Perth, on Friday last. The meeting of all times and climes made an unusually brilliant scene, and the dance, an unqualified success, was heartily enjoyed by the patients. An interesting part of the proceedings was the presentation to the retiring Matron, Miss Sharp, of addresses, one from the nurses, and one from the lady patients in the institution.

THE GOLDEN APPLE.—There were some twenty to thirty nurses present at the representation of this charming and amusing opera at St. George's Hall. It was a great pity more nurses had not availed themselves of the chance, for the music was excellent, light, and tuneful, and the piece well put on the stage. The parts of Venus and Thetis were played with particular merit, while Hebe looked charming and had the most becoming gown. It is seldom that an amateur performance is so thorough as was the representation of "The Golden Apple," and we congratulate all concerned. The hall was well filled, so we hope a nice sum was secured to help Miss Marsden on the difficult and dangerous path she is now treading. She needs money, she cannot succeed without it, and surely if hers is the peril ours should be the expense.

ATCHAM AGAIN.—It was our sad duty in July, 1889, to chronicle the fact that the Guardians of Atcham Union deliberately appointed imbeciles as nurses in the Infirmary. The Local Board took up the subject, and impressed on the Guardians the ignorance and evil of their course; the result was the institution of three trained nurses about twelve months ago. But Atcham men appear to suffer terribly from economy towards the sick poor, and on the first Saturday of the new year Mr. H. Healing moved a resolution to the effect that only two paid nurses be employed in the hospital at the workhouse, and that all resolutions to the contrary be rescinded. He was quite sure that two nurses were all that were wanted at the Union. He would be very sorry for any poor person to be neglected, but he did not consider that there was any need for a night nurse. He did not see why the nurses could not go round the hospital the last thing at night and the first thing in the morning, and if they happened to be wanted during the night there was always someone to call them up. He considered that they had amply proved that three nurses had never agreed, and as long as they had that number something would always be cropping up. He also considered that the Infirmary was worked at a very great expense. In answer to Mr. Healing, the Master stated that there were about 100 patients in the hospital. Dr. Cureton said he considered it to be absolutely necessary for them to have three nurses, one fully qualified and two partially qualified. The Rev. A. G. Kingsford said he was sorry to have to differ with Mr. Healing, but still at the same time he must say that he considered that the hospital management under three nurses had been a great success. It was only by thirteen votes to nine that Mr. Healing's resolution was confounded. Taking this story, together with the story of the Brentford Guardians we told a fortnight ago, we feel inclined to join the prayer of the contumacious old pauper "That Providence would be pleased to soften the hearts of all guardians, or else give 'em all the rheumatics."

LIVERPOOL NORTHERN HOSPITAL.—A member of the nursing staff of this hospital was lately married to Mr. R. C. Kerr, and that gentlemen has just given £100 to purchase a library of books for the use of the nurses. This is an excellent movement, and it looks as though Mr. Kerr were grateful for the wife the institution has conferred on him.

MRS. ERNEST HART.—On Tuesday, at the Princes' Hall, Mrs. Ernest Hart, one of the most energetic of lady philanthropists, delivered an interesting lecture on "Industrial Development and Technical Teaching in the Wilds of Donegal." Mrs. Hart is a graceful and earnest lecturer, and her words were illustrated by some excellent photographs. The work she has started in Donegal is deserving of all support.

AN INSTITUTE FOR AYLESBURY.—Mr. Jowett has raised the proposition that an institution for trained nurses is needed at Aylesbury, and could be attached to the Bucks Infirmary. Mr. Walter Hazell and others second the idea. If possible, the gentlemen of Aylesbury want to combine district nurses and private nurses. We would suggest that the district nurses be quite distinct and have a home of their own; whereas the private nurses could be attached to the Infirmary. The difference between a district nurse and a private nurse is not always so plain to the public as we could wish.

SHORT ITEMS.—Miss Marie G. Magill, of Melbourne, has been complaining in public of the overwork of the nurses of the Alfred Hospital.—*Truth* is attacking St. Bartholomew's because of the number of nurses ill there. It is not likely that Sir Sydney Waterlow and others, who have ever done their best for nurses, would let abuses exist if they knew a remedy.—Several of the nurses of the Royal Free Hospital attended the Lord Mayor's reception on Tuesday in aid of that excellent institution.—We understand that Miss Napier, of Winchester, who recently went to St. Thomas's, was appointed to the "extra" staff, which has always been open to nurses other than Nightingale trained.

LIVERPOOL JUBILEE INSTITUTE.—The inaugural festival of this institute was held last week, Mr. Charles Langton in the chair. Mr. and Mrs. Gilmour, Miss Rosalind Paget, Mr. S. G. Rathbone, Dr. Paull, and others were present. The Chairman, in opening the meeting, referred with regret to Mr. Rathbone's absence, and said that gentleman had looked forward with great pleasure to attending. He really was the founder of their training school, and had for thirty years given them great support. The badge of the institute for long and good service was conferred on Miss Stains for 23 years' service (unfortunately she was absent owing to illness), on Miss Cook for 23 years' service, and on 4 other Matrons and 16 other nurses. The badge can only be retained so long as the nurses continue to work for the Jubilee Institution. Subsequently there was a musical entertainment by Mr. M. B. Spurr, and a performance of the farce, "Ici on Parle Français," in which Mrs. Moore, the Misses Rathbone, and Messrs. Edmond Rathbone and Arthur Rathbone took part. Supper closed a very pleasant evening, for which the nurses and the other members of the audience were indebted to the kindness of Mr. Rathbone.

### The Nurses' Co-operation.

When Kingsley, in middle-age, returned from the tropics, which he had written about but never seen in his youth, he sat down and wrote an account of his travels under the title of "At Last!"

That exclamation must have been on the lips of many nurses, on Tuesday, when the first general meeting of the Nurses' Co-operation was held; longed for for years, planned eighteen months ago, and worked out with might and main all through the autumn and winter, the cherished scheme at last has taken visible form and shape. It is worth while to trace its progress. In The Hospital for November 9th, 1889, appeared a letter on "A Co-operative Registry Office for Trained Nurses," written by Miss Belcher, a private nurse, holding the L.O.S. diploma, who chose, for the time being, to pass under the pseudonym, "One of Many." She pointed out that a central office would be a great boon to the many private nurses working on their own account, and gave the names of five nurses willing to combine to start such an office, if the Hospitals Association would help them. The Hon. Secretaries of the Hospitals Association willingly met Miss Belcher and her friends, and as the scheme seemed feasible, a small provisional committee was formed. This committee had the help of such noted nursing authorities as Mrs. Dacre Craven, the oldest trained nurse in England save Miss Nightingale, and who has had even wider experience than Florence Nightingale can boast; and also the advice and support of Miss R. Napper (Sister Edith), for many years Matron of the Princess Alice Hospital at Eastbourne.

Mr. P. Michelli from the first kept an eye on the business part of the proceedings, and perpetually damped rash enthusiasm by a habit of "doubling the expenditure and halving the receipts" before he would deal with any of the figures submitted to him. Dr. James Goodhart was also an early friend of the movement.

The late Countess of Rosebery very kindly and very carefully considered the scheme, and presided at one or two of the committees. The Co-operation had hoped to have her name as the first president of the society, and her loss here, as in all departments of the nursing world, has been keenly felt.

The work of the provisional committee was difficult. Particulars of the pay given by fifteen nursing institutions were procured; all the papers and blanks of the Philadelphia Directory were obtained, and gradually the scheme began to take definite form. Substantial help and valuable time were given to the co-operation by those who prefer to remain nameless, but their kindness can never be forgotten by the nurses who benefit by the central office.

The question of Lady Superintendent arose to puzzle the Committee, and this difficulty was only solved when Miss Philippa Hicks, who was then leaving the Hospital for Sick Children in Great Ormond Street, came forward and offered to take the post. Miss Hicks had made the private nursing institution at Great Ormond Street a great success, and had arranged for the nurses there to take a percentage on their earnings. She was also a Nightingale nurse, had been Superintendent to the National Aid out in Egypt, and home sister at King's College Hospital. Here, then, was the very woman for the post. The next step was to take offices, and they were secured at 8, New Cavendish Street, Portland Place, W., a delightfully central situation. Then came the formation of a general committee, and surely there must have been something specially attractive to law and justice in the scheme, from the number of our warm supporters who are solicitors: Mr. Charles Cheston, Mr. Ayerst Hooker, Mr. H. Kimber, M.P., and Mr. Capel Slaughter! Sir William Moore, Dr. Hadden, Mr. Howard Barrett, Mr. Charles Keetly, Mr. Bernard Pitts, and others came forward to represent the medical profession, while Miss Jane Wilson, Mrs. Malleson, and Sister Katherine, of Plaistow, represented other nursing associations. Such is the history of the movement; the result is the following scheme:—

### The Nurses' Co-operation.

Lady Superintendent: Miss K. Philippa Hicks.

Offices: 8, New Cavendish Street, Portland Place, W.

A society incorporated under the provisions of the Companies Act of 1862-90, pursuant to which none of the society's profits can be distributed except among the nurses who join the society.

Candidates for the register must be approved by the Lady Superintendent and enrolled by the Committee.

No nurse can be admitted to the register unless she hold a hospital certificate, or can produce satisfactory evidence of one year's hospital training directly followed by two years of private or district nursing. In the latter case facilities will be offered to nurses to pass an examination before an approved Board of Examiners. Evidence must also be supplied as to moral character, state of health, and place of residence.

Each nurse will, at the termination of her case, be paid the full amount of her earnings, less $7\frac{1}{2}$ per cent., which will be placed to a common fund, out of which the yearly working expenses of the co-operation will be defrayed. The accounts of the co-operation will be made up annually to December 31st and audited by a professional accountant. Any surplus of the common fund then remaining will be available for distribution amongst the nurses as may in general meeting be determined.

Nurses arrange their own fees, but the minimum charge is one guinea a week.

The object of the co-operation is not solely for the supply of private nurses, but for their general benefit. It is proposed immediately to start lectures and clinical instruction for the nurses, and in this direction several medical men and Matrons have offered their services.

The management of the co-operation is vested in a General Committee, twelve members of which will be appointed as an Executive. At least six nurses shall be on the Executive Committee.

At the general meeting on Tuesday, Mr. Charles Cheston, Chairman of the East London Hospital for Children, was in the chair, and was elected permanent Chairman of the General Committee. Mr. Cheston made an admirable statement of the aims and hopes of the Co-operation, stating that he could not consider involuntary subtractions from a private nurse's earnings as proper voluntary subscriptions to a hospital. The Executive Committee of the Co-operation was then elected, Dr. James Goodhart being elected Hon. Treasurer. The following resolution was unanimously carried: "That the thanks of the meeting are due to Mr. Capel Slaughter and Mr. Henry C. Burdett for the active interest they have taken in the Co-operation, and that if the articles of association permit they be elected as Vice-Presidents of the Co-operation." When business was over, tea was provided in the nurses' sitting-room, and the premises were open to inspection. Several of the nurses who have joined the Society were present in uniform.

The Co-operation desires to try and work in harmony with all existing institutions; it recognises that especially those institutions in connection with hospitals have great advantages, but it also recognises that there are many nurses who only work well when they have to make their score off their own bat. Each nurse can surely find her own sphere now; she can have her certain income from an institution, and the absence of all wearing responsibility, or she can take her share of risk and comparative freedom as a member of the co-operation. Any nurses desiring further information about the co-operation should call at the offices between ten a.m. and four p.m. (Saturdays, ten to twelve noon), or on Thursday evenings between eight p.m. and nine p.m.

### Nursing Medals and Certificates.

### THE BUTTERWORTH MEDAL.

THE medal which we illustrate to-day is that given to the nurses at Guy's Hospital who complete five years of honourable service. The medal was instituted in 1890 by Mr. J. W. Butterworth, and consists of a silver order surmounted by a safety pin. On one side of the medal is the portrait of Thomas Guy, copied from a picture belonging to Mr. Butterworth; on the other side is the name of the recipient nurse. Once won the medal remains the property of the nurse. The Matron of Guy's, and Sisters Patience, Cornelius, Clinical, and Grace all wear the medal, The certificate at Guy's is given to paying probationers at the end of one year, to non-paying probationers at the end of three years.

On January 7th, 1890, the following nurses received the medal:—

| | |
|---|---|
| Elizabeth Newman, 26 years. | M. Botwright, 5 years. |
| Margaret Keogh, 24 years. | Beatrice Harding, 5 years. |
| E. Butler, 17 years. | Ellen Mayne, 5 years. |
| H. C. Gore, 20 years. | Annie Moorcroft, 5 years. |
| Victoria Jones, 10 years. | Helen Peckham, 5 years. |
| J. W. De Wet, 9 years. | M Rawlison, 5 years. |
| Phyllis Phillips, 9 years. | Fanny Simms, 5 years. |
| Caroline Stevens, 9 years. | Maud Wilks, 5 years. |
| Grace S. Benson, 6 years. | Louisa Wiskin, 5 years. |
| Florence Bower, 6 years. | |

This January twelve nurses concluded the necessary term of service and received the medal:—

| | |
|---|---|
| Elizabeth Crisp. | Hannah Morris. |
| Agnes Sims. | Annie Whebell. |
| Mary Agnes Clarke. | Charlotte G. Rodwell. |
| Mary Ashford. | Nellie Strick. |
| Theresa Caroline Steer. | Charlotte L. Still. |
| Lucy Ann Rogers. | M. A. Barker. |

### Presentations.

MISS WELLS, the lady superintendent of the Jersey Institute, was the recipient of a handsome silver toastrack on Christmas morning from her nurses as a small token of their esteem.

ON Christmas Day the nurses of Her Majesty's Hospital, Stepney Causeway, E., presented the matron, Miss A. Warburton (Sister Sara), with a very handsome marble clock as a slight token of gratitude for all her past kindnesses to them, and as an indication of best wishes for her future welfare. Miss Warburton felt the value of the gift much enhanced by the nicely-worded address accompanying it, expressive of the good feelings of the nurses, and signed by them all.

THE Council of the Royal College of Physicians, Edinburgh, on the recommendation of Dr. Clouston (Morison lecturer for the year) has awarded the prizes left by the late Sir Alexander Morison, a former President of the College, for meritorious attendance on the insane, to Mary Anne Anderson, Midlothian and Peebles Asylum, and to David Robertson, James Murray's Royal Asylum, Perth. The former has seen eleven and the latter thirty years' service. We congratulate Scotland on having a fund for so laudable a purpose, and the fortunate two whose faithful services have been so fittingly rewarded.

MISS LANDALE, Matron of the Fleming Memorial Hospital, Newcastle, has been presented by her sisters and nurses with a handsome inkstand with inscription.

### GRATITUDE.

THE heart's true happiness and comfort follows on love to God, and we shall find it is for our earthly as well as spiritual benefit if we love Him with all our hearts. Unfortunately we are too often prevented by ingratitude. We are like a person who has had a great kindness done to him, in fact so great a one that nothing can repay it. He hates the feeling of being under an obligation, so makes light of the benefits he has received, declares they were not so very great after all, and repeats the fiction till he ends by believing what he wishes was the fact. Do not we feel angry and disgusted at such conduct? And yet this is just the way we act towards God. Our Heavenly Father has done so much for us that we tire of acknowledging His benefits. We want to forget that we are His by right, because He made us, and has, therefore, a claim on our gratitude and obedience. But how kind, how long-suffering He has been with us sinners! I am sure if you or I had made a toy, or a piece of machinery for our pleasure, and found that it would not act as we expected it to do, and even vexed and annoyed us by doing the exact opposite to what we wanted it to do, that we should have thrown it away in our anger and cared nothing for what became of it afterwards. How differently our Heavenly Father has acted towards us! He made man for His own pleasure and glory, and when he fell through disobedience, instead of destroying the whole race, God immediately gave them the promise of a Saviour who should restore them to their first estate. When the fulness of time was come He sent His Son to invite the wanderers back into His arms. He calls them by every endearing name. They are His sheep, whom He seeks to save, His prodigal sons, whom He loves so dearly, His lost piece of money which shall be sought for until it is found. Who can resist such loving entreaties? Come back, ye wanderers, to the fold: here is green pasture for the hungry, and cool, pleasant streams for the tired and thirsty. Come back, oh prodigal, leave the husks and the swine, all that is left of sinful pleasures. You are poor, and naked, and miserable, but your loving Father has already killed the fatted calf, brought out the robe and the ring, and the shoes for your feet, and seeing you a long way off runs to meet you. Do not be ungrateful, let Him fall on your neck and kiss you. Do not forget the portion He gave you at first, and which you have squandered. For past and present mercies let your gratitude and joy rise to Him. Let us gain true happiness, then, by receiving the blessings He showers upon us with gratitude and love. It is only the noblest minds which can receive an obligation gracefully. We will try to have noble minds, and, with grateful hearts and cheerful voices, exclaim both by word and action, Praise thou the Lord, oh, my soul, and all that is within me praise His holy name, and forget not all His benefits.

### Medical Applications of Electricity.

A very interesting lecture to nurses on the above subject was given at the Institute of Medical Electricity in December by Mr. H. Newman Lawrence, M.I.C.E. The lecturer commenced by stating that he was not a medical man, and did not wish to be considered as such in any sense. He appeared there as an electrician, or rather as an electrical engineer having to apply his engineering to the most wonderful machine ever constructed, viz., the human body. He also claimed kinship with the honourable profession of his audience in so far as his occupation consisted in carrying out the wishes and instructions of medical men upon their patients.

Mr. Lawrence proceeded to refer to the fact that electricity is the same, however generated, in so far as it will perform the same chemical, mechanical, thermal, and physiological actions; but that it could be made to assume different forms by various transformers, such as the induction coil interposed between the generator and the portion of the circuit where the work is required to be performed. Differences in the relative value of the factor, electro-motive force, and circuit-strength were thus produced, which were capable of performing fresh actions, physiological and otherwise. The main differences between the continuous, interrupted, and alternating currents were briefly described, and then, by the aid of experiments and numerous diagrams, some of the chemical and physiological actions of electricity were shown. Electrolysis, cataphoresis, and catalysis were referred to as most important actions when dealing with electrical applications to the body, and the fact that these are mainly proportional to the current-strength employed was strongly urged. The resistance to electricity of the human skin was shown to be very considerable; a list of measurements of resistance of ten different persons was exhibited, from which it was seen that normally dry skin offered great resistance to both continuous and alternating currents (coil); that this resistance could be much reduced by moistening the skin under the electrodes; but that both when dry and when moist the resistance to continuous current was far greater than that to alternating current.

The lecturer then said: We now come to the important question of dosage. I am sorry to say that this is at present an uncertain and indefinite matter with many medical men. Definite and accurate terms are absolutely necessary for dosage. It is an utter fallacy to measure dosage by cells. It should be defined in terms of current strength and time. Without distinct declarations on these two points, it is impossible to know what to do. The current strength is important, as it is the factor which produces electrylosis and cataphoresis, and it is the only factor as regards their production, but as regards their continuation we must have a measurement of time. Hence the necessity of defining the doses of electricity by current strength and by time. The standard of measurement of current strength adopted by electrical engineers is the ampère; but this is far too great for medical purposes. One ampère is more than can be safely used, even under an anaesthetic. As the ampère is too large, the thousandth part of it, called the milli-ampère, has been adopted for medical purposes. As I have said, it is no use prescribing so many cells and trusting to luck as to the amount of electricity that passes through the patient. We must prescribe exactly in milli-ampères and minutes.

There is another thing which some people are fond of trusting in, but which we must never rely upon as a measurement of electricity, and that is the sensations of the patient. We must not trust to the answers to the questions, "Does it hurt?" or "Do you feel that?" as sensation is most misleading. The patient's sensibility may be affected either in the way of being abnormally acute or abnormally dull. What we have to do is to give a definite quantity, no matter what the sensation may be. But remember that this does not mean that we are to give the patient pain. At first patients are always nervous, and therefore we should be careful not to frighten them. We must not be frightened ourselves. A nurse who is herself afraid of the battery should not attempt to electrify a patient. Having acquired a proper knowledge of the agent we employ, and its various peculiarities, we must put on a bold face, and inspire confidence in our patient. There is a definite thing to do with a carefully measured quantity, and it is only by doing it calmly and carefully that accuracy can be arrived at and satisfactory results obtained." In illustration of the absurdity of prescribing electricity by naming a definite number of cells, the lecturer tried fifteen cells of a Leclanche battery, each having an E.M.F. of nearly $1\frac{1}{2}$ volts, and found by applying electrodes of different degrees of moisture connected therewith to a subject, that different current strengths were obtained varying in so great a proportion as 1 to 3.

*(To be continued.)*

### Keeping Christmas.

The Christmas festivities of **Her Majesty's Hospital** extended into the New Year. Christmas Day was very pleasantly spent, the wards and corridors having been beautifully decorated with appropriate mottoes in most æsthetic colours. Every patient having received a present in the morning, all who were convalescent and able were regaled with a good dinner of roast beef and plum pudding. The chief treat of the season, however, consisted in a special entertainment on the 6th inst., provided through the kindness of Mrs. B. A. Miller, who, with several friends, arrived at the hospital early in the day, and, as the result of their labours, a beautiful tree (given by Mr. T. Fowell Buxton), laden with handsome presents, delighted the eyes of as many of the patients as were well enough to be placed together in the Gordon Memorial Ward. Each received more than one present, and a new silver piece of money. Before the giving out of the presents a first-class conjurer delighted the audience with his clever tricks, immense approval being shown by numerous bursts of applause. At the close of the entertainment the patients expressed their appreciation of the kindness shown to them by their hearty cheers given to the ladies, and also to their medical officer, Dr. R. Milne.

At the **Wolverhampton Infectious Hospital** a dinner was given to the staff, followed by a social evening. Mr. Franks proposed "The Health of the Matron (Mrs. Chambers)." A farce was acted by five of the nurses, Mr. Franks gave some recitations, and Mr. Hodges, Mr. Hill, and others sang. The evening was greatly enjoyed by all.

The patients in the **Portsmouth Hospital** had their annual Christmas tree, organised by the Committee of Management. There were presents for everybody, both patients and visitors, and after the stripping of the tree there was an excellent concert, followed later on by a dance for the nurses. There was a large attendance of subscribers and their friends.

A very happy season has been spent by the little ones at the **Fleming Memorial Hospital**, and many treats prepared and carried out. Besides the orthodox Christmas fare, a large Christmas tree was provided in each ward, laden with toys to gladden their eyes and hearts, and parcels of warm clothing to wrap their small limbs in on leaving hospital. Choir boys came and sang carols, also a trio of German zither players and a violin party entertained on other afternoons. A pleasing and much-enjoyed feature was an extra special visit of the parents and friends, who had tea with their children, and on yet another evening former patients came to enjoy their share of the good things. Of course, the time-honoured Punch and Judy came to pay their respects to the patients, and with a magic-lantern to wind up, we think the children had what Americans call "a good time."

At the **Gravesend Hospital** the patients had a real old-fashioned Christmas, and greatly enjoyed seeing the residents and the nurses rushing hastily to their bedsides with pudding all alight. The crowning treat of tobacco was also permitted. The children had presents put in their stockings. On Twelfth Night a dramatic entertainment was given and everything was done with as much thoroughness as though royalty was present. Afterwards the members of the Ladies' Committee presented gifts of warm clothing to all the patients. On the 7th the nurses had a musical evening, and hearty thanks were felt to be the due of the Matron and all concerned in providing such seasonable cheer.

At **Carmarthen Infirmary** an enjoyable entertainment was given in the prettily decorated corridor. After some songs, the farce "Ici on Parle Français" was admirably acted. The people of the town, the Matron, and the nurses all did their best to give the patients a pleasant time.

Thanks to the forethought of the people of Boston and the efforts of Miss K. M. Heanley, the patients at **Boston Hospital** were able to spend a very pleasant Christmas. At eleven o'clock there was service, followed by carols, and in due course came the usual Christmas dinner. The hospital was looking its very best, in spite of the fact that the matron was just changing probationers. Mr. Fred Frank has lately given £100 to this hospital.

At the **Nottingham General Hospital** Mr. Tresider and Miss Rimington worked hard to make the Christmas tree a success, and there was a goodly attendance of visitors from the town.

### Everybody's Opinion.

*[Correspondence on all subjects is invited, but we cannot in any way be responsible for the opinions expressed by our correspondents. No communications can be entertained if the name and address of the correspondent is not given, or unless one side of the paper only be written on.]*

### NURSES AND INFECTION.

SIR,—Allow me to bring before your notice the following facts; deal with them in THE HOSPITAL as you deem best. It is not from any vindictive feeling I write you, but simply to save other families a similar sorrow and to spare if possible other valuable lives.

On or about November 7th or 8th, 1890, Nurse Bishop went to Paddington Green Children's Hospital to oblige by taking duty until a nurse was engaged, the previous night-nurse having been taken away same day ill. Nurse Bishop was put into same room, bed, and bedding (save the sheets). A few days afterwards she complained of headache, also of high temperature (101) to her sister, and mentioned the fact of being put into same bed and bedding. Anxious to keep up she remained on duty until she fell in a dead faint during the night, cutting her head and bruising her body. Her fellow night-nurse called assistance. Nurse Bishop was put to bed. The following morning she wished to go home to "Tooting," and was allowed to do so unattended; arrived there in a state of utter exhaustion; was put to bed; carefully nursed; following day temperature of 103. She died on December 9th of typhoid after three weeks' illness. (*See* HOSPITAL, December 27th.)

Case No. 2, same hospital.—Nurse Phillips (Alice Emma, in her 20th year) resigned her post on December 22nd, 1890, not having felt well for some time. Upon arriving home took to her bed same day, and died in eleven days of typhoid fever with chest complications. Temperature ranged from 101 to 104·6 in this case; delirium so bad no information could be obtained. Now, sir, I would ask you who is responsible for this state of things? Two precious lives lost which probably could have been saved by timely observation and care.

I am given to understand the three nurses quoted above had occupied the same room. What is the sanitary condition of this institution? Trusting you will afford space in your valuable journal to ventilate this matter.—I am, yours faithfully, E. J. PHILLIPS,

Matron, Norwood Cottage Hospital,
Hermitage Road, Central Hill, Norwood, S.E.
January 12th, 1891.

SIR,—In answer to the charges brought against us by the Matron of the Norwood Cottage Hospital, I can only say that we consider we have shown neither neglect in the care of those nurses mentioned by her, nor have we any reason for thinking that the sanitary condition of the Hospital is at fault. Any further enquiries which may be deemed necessary I shall be pleased to answer. E. A. ANDERSON, Matron.

Paddington Green Children's Hospital,
January 19th, 1891.

### THE PENSION FUND.

"L. W." writes: May I, as a private nurse, be allowed, through the medium of THE HOSPITAL, to express my thanks, and I am sure I may add those of many other private nurses, to the Council of the Pension Fund for the kind and valuable concessions which they have made to us in allowing us from henceforth to use the fund as a Savings Bank, and pay in irregularly as it suits our purposes? It is a great boon to the private nurse, who one month may be rejoicing in a comfortable income, and the next deploring an increased outlay! I trust many of my fellow-workers may follow the example of the writer, and make up their minds to pay in a little extra to increase their pensions according as God prospers them. We need not now (what I feared at one time to be inevitable) tie ourselves to a small pension for fear of robbing the poorer members of our families of the help which so many of us habitually give.

### HELP WANTED.

"E. D." writes,—I wonder if any of your many readers could be of assistance to a lady friend of mine who is anxious to keep her home over her head, and could easily do so if able to earn four or five shillings weekly with morning or afternoon engagements, such as reading to invalids, making their purchases, chaperoning children to and fro to their masters, entertainments, or in their walks. In an emergency she would be extremely useful as housekeeper or companion, and has the advantages of having moved in a good social position, is well educated, bright, and cheery. Some years ago she was deserted by her husband when quite young, and left to face the world with two children, a boy and girl, the eldest being only six years of age at the time. After endless struggles she has managed to educate and place them in life, and now, unfortunately, through no fault of their own, are able to maintain her only partially. My statements can be verified by the referees, whose names are enclosed, who have all known her for some years. I shall be pleased to give her address to any of my fellow nurses or their friends, and take the liberty of thanking you in advance for the publicity I feel quite sure you will give the case through the medium of your valuable paper.

### FEVER HOSPITALS.

"SINCERITY" writes: I was pleased to see in your number of December 20th, that you had a kind word to say for the North-Western Hospital and its nurses. I have often felt that the hospitals and nurses under the Metropolitan Asylums Board were left out in the cold. Why are they overlooked? With reluctance I must answer myself, because we cannot compare with other hospitals, and we never shall be able to hold up our heads with them as long as ward servants are promoted to the nursing staff, and as long as untrained people are employed to superintend. But the Metropolitan Asylums Board seem to have become conscious of these facts; and it is good to see how some of the hospital authorities under this Board try to secure the services of trained Matrons and trained nurses. Others like the old way best, but they will soon have to follow suit; they will be obliged to get trained nurses, not for the love of them, but because it is the fashion. I am looking forward to a time when our editor will be able to speak of the hospitals and the nurses under the Metropolitan Asylums Board as proudly as he speaks of the Grand Old London and its excellent Matron and faithful nursing staff. I hope, with Miss Anderson, that there will be a thorough investigation at Homerton, and I hope it will end with better results than the inquiry that was made at that hospital a few years ago.

### "IN DARKEST ENGLAND."

"Brisbane" writes: I have just read "General" Booth's book, "In Darkest England," and have come across two passages which I think ought not, in justice to the hospitals, to be passed over without comment. On page 55, under the heading of "The Woolwich Dust-hole: Sickness, Friendlessness, Death," he says, apropos of the unfortunates, "In hospitals it is a known fact that these girls are not treated at all like other cases; they inspire disgust, and are most frequently discharged before they are really cured." Is not this a libel? I could name one large West-end hospital to which one of "these girls" was brought straight from the street. What treatment did she receive? Was she neglected, as Mr. Booth would have us believe? Far from it. Her case was a most critical one, and two special nurses were engaged from a nursing institution—one to nurse her by night, and the other by day—at a cost to the hospital of one guinea a week for each nurse, besides their food. In our largest London lying-in hospital, where I was trained, and in which, when I was there, two-thirds of the patients were single girls, a nurse was liable to summary dismissal if it was known that she in any way taunted these girls with their sin, or treated them in the slightest degree differently to the married women. And I believe all hospitals to be alike in this respect, notwithstanding "General" Booth. The second paragraph will be found on pages 220—221. It runs thus: "In cases of sickness he (the poor man) can apply to the parish doctor or the great hospital, to receive an odd word or two of advice, with a bottle of physic, which may or may not be of service." "General" Booth does not include a hospital in his scheme, but if these passages show his estimate of existing ones, the sooner he starts one the better for his people, who at present, I suppose, are obliged to be content in ill ess with those he condemns! If you think these remarks worth insertion in The Hospital pray use them.

### Notes and Queries.

**Answers.**

(23).—Apply to Nurse Barrett, Hazlewood, Near Derby, or to Cottage Hospital, St. Paul's Cray, Kent, or to Home for Invalids, Grange Mount, Birkenhead, or to 8, Clepstone Avenue, Nottingham.

(26) If A. G. Farrer will communicate with G. H. A. at Stratton Cottage, Frith Hill, Godalming, no doubt arrangements can be made for the reception of the gentleman referred to in the home of a trained nurse.

*H. M.*—Nurses can wear gloves when in uniform if they are at a dance or theatre, or evening entertainment. Light gloves should be worn, either white or tan.

### Amusements and Relaxation.

### SPECIAL NOTICE TO CORRESPONDENTS.

First quarterly word competition commenced January 3rd, 1891; ends March 28th, 1891.

Competitors can enter for *all* quarterly competitions, but no competitor can take more than *one first prize* or *two* prizes of any kind during the year.

Three prizes of 15s., 10s., 5s., will be given for the largest number of words derived from the words set for dissection.

Proper names, abbreviations, foreign words, words of less than four letters, and repetitions are barred; plurals, and past and present participles of verbs, are allowed. Nuttall's *Standard* dictionary only to be used.

N.B.—Word dissections must be sent in WEEKLY not later than the first post on Thursday to the *Prize Editor*, 140, Strand, W.C., arranged alphabetically, with correct total affixed.

The word for dissection for this, the FOURTH week of the quarter, being "ICEBERGS."

| Names. | Jan. 15th. | Totals. | Names. | Jan. 15th. | Totals. |
|---|---|---|---|---|---|
| Reynard | 47 | 77 | H. A. S. | 42 | 68 |
| Reldas | 46 | 76 | Sister Jack | — | 26 |
| Tinie | — | 30 | Crystal | 44 | 70 |
| Patience | 46 | 76 | Woodbine | — | 25 |
| Jenny Wren | 42 | 72 | Madame B. | — | 25 |
| Agamemnon | 46 | 76 | Shakespeare | 35 | 59 |
| Wyamaris | 44 | 73 | Smyrna | 20 | 43 |
| E. C. | 46 | 75 | Southwood | 22 | 43 |
| Ecila | 43 | 72 | Gipsy Queen | — | 21 |
| Hope | 46 | 75 | Snowball | — | 19 |
| M. W. | 46 | 75 | Rita | 20 | 36 |
| Qu'appelle | 47 | 75 | Mortal | — | 16 |
| Nil Desperandum | 47 | 75 | Nurse Annie | — | 15 |
| Lady Betty | 46 | 74 | Carmen | — | 11 |

**Notice to Correspondents.**

N.B.—Each paper must be signed by the author with his or her real name and address. A *nom de plume* may be added if the writer does not desire to be referred to by us by his real name. In the case of all prize-winners, however, the real name and address will be published.

### A Winter Holiday.

### II.—ACROSS THE CHANNEL.

Before beginning our foreign travels, we two nurses had made a grand resolution, which proved easier to make than to keep. We had decided, Frances and I, that we would leave our ordinary working lives behind us, and when we crossed the Channel the hospital should not cross with us. We would forget the wards! We would dismiss from our thoughts the sadness, the "unloveliness" of the great mass of suffering humanity with whom we had cast in our lot for these many years past. Yes! we two women of experience actually sat and plotted to do this, just as calmly as we planned the two simple travelling dresses with which we replaced, for the time, our nurses' uniform. And how long did we maintain this most virtuous resolution? During the first two days of our holiday, that part of our travels which found us least inclined for conversation of any kind, we were so weary then, and there was so much excitement, too, attending our entrance into the (to us) new world. But after these first days our conversation frequently drifted homewards, and from the Pyrenees to Whitechapel was a journey traversed rapidly by thoughts and tongues. We retained sufficient respect for our foregone resolution to consider it proper to reproach each other for this "breach of contract," but I soon noticed that the reproof was carefully delayed until the speaker had finished her hospital references. Then came the prim "I thought we were not to talk shop!"

How could we help it when each fair sight and sweet scent brought to remembrance those who could share none of these beauties. So a gay Spanish shawl was purchased for one sick person, and a root of maidenhair fern, packed in a tin box and posted to another old woman who had been "bed-ridden" for many years. I found it growing and flourishing under her tender care months after we had settled again to our work.

By the way, it used to puzzle me once to understand why a poor man or woman should so frequently, when elderly, become "bed-ridden." A wider knowledge of the poor and their homes has shown me how much less uncomfortable the invalid is in the bed, of which his possession is undisputed, than if he feebly inhabits one of the limited number of chairs in the cramped quarters where "the family" lives. Also a distinction is conferred by the neighbours on a person who "has kept his bed for years," in which other members of the circle indirectly participate.

But to return to ourselves, many were the funny little events and trifling accidents which we laid by in our minds for the benefit, on some future day, of fellow nurses who had, like ourselves, that sense of humour which helps so bravely many of those whose lives are spent in serious scenes. It seemed as if we could in this way, enable them to share our enjoyment, although but to a limited extent. So we drifted on in chats and thoughts, revelling not a little in the feeling of being perfectly "rested," which is a sensation enjoyable from its rarity in the life of a nurse. We get so used to being tired, that until the fatigue oversteps an endurable limit we hardly think the condition needs remark, least of all amongst companions who are just as weary as oneself.

(*To be continued.*)

## Figures and Tables

**Figure f1:**
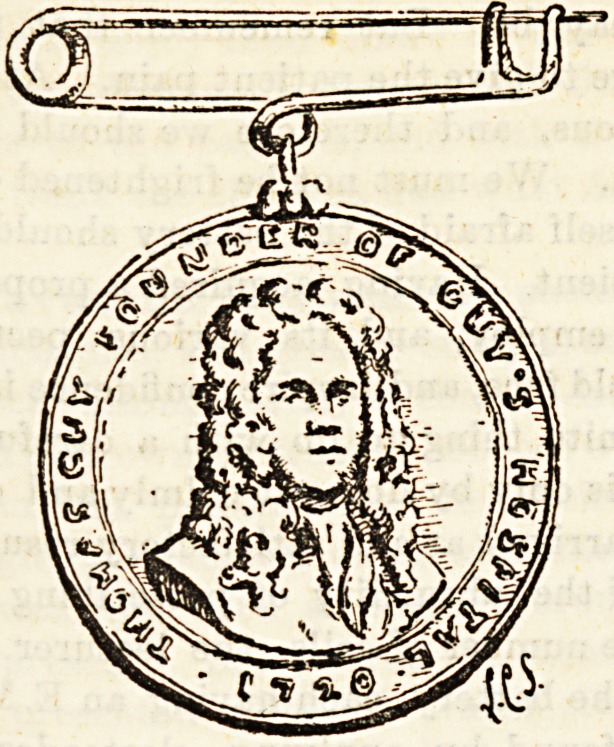


**Figure f2:**